# Workgroup Report: Indoor Chemistry and Health

**DOI:** 10.1289/ehp.8271

**Published:** 2005-11-03

**Authors:** Charles J. Weschler, J.R. Wells, Dustin Poppendieck, Heidi Hubbard, Terri A. Pearce

**Affiliations:** 1 International Center for Indoor Environment and Energy, Technical University of Denmark, Lyngby, Denmark; 2 Environmental and Occupational Health Sciences Institute, University of Medicine and Dentistry of New Jersey and Rutgers University, Piscataway, New Jersey, USA; 3 National Institute for Occupational Safety and Health, Morgantown, West Virginia, USA; 4 Environmental Resources Engineering, Humboldt State University, Arcata, California, USA; 5 Department of Civil Engineering, University of Texas–Austin, Austin, Texas, USA

**Keywords:** allergies, asthma, biomarkers, environmental cancer, free radicals, hydroperoxides, indoor pollutants, inhalation exposure, lung damage

## Abstract

Chemicals present in indoor air can react with one another, either in the gas phase or on surfaces, altering the concentrations of both reactants and products. Such chemistry is often the major source of free radicals and other short-lived reactive species in indoor environments. To what extent do the products of indoor chemistry affect human health? To address this question, the National Institute for Occupational Safety and Health sponsored a workshop titled “Indoor Chemistry and Health” on 12–15 July 2004 at the University of California–Santa Cruz. Approximately 70 experts from eight countries participated. Objectives included enhancing communications between researchers in indoor chemistry and health professionals, as well as defining a list of priority research needs related to the topic of the workshop. The ultimate challenges in this emerging field are defining exposures to the products of indoor chemistry and developing an understanding of the links between these exposures and various health outcomes. The workshop was a step toward meeting these challenges. This summary presents the issues discussed at the workshop and the priority research needs identified by the attendees.

The National Institute for Occupational Safety and Health (NIOSH) established the National Occupational Research Agenda (NORA) in 1996 with input from more than 500 organizations and individuals. The indoor environment was included in the 21 NORA priority areas, and the NORA indoor environment team was established. Its goals included focusing and facilitating research that would improve the health of workers in indoor environments.

To address causes and prevention of specific building-related health effects, the NORA indoor environment team conceived and sponsored a workshop titled “Indoor Chemistry and Health.” Indoor chemistry is defined as reactions involving indoor pollutants, occurring either in the gas phase or on surfaces. In the absence of combustion, such chemistry is often the major source of free radicals and other short-lived reactive species in indoor environments. Approximately 70 scientists from eight countries participated in this workshop held at the University of California–Santa Cruz on 12–15 July 2004. Disciplines represented included atmospheric chemistry, chemical engineering, toxicology, medicine, epidemiology, architecture, and public health. [A full participant list can be viewed at the NIOSH NORA indoor environment website (Centers for Disease Control and Prevention 2005.]

A major goal of the workshop was to promote communication between persons examining the health effects resulting from exposures to airborne pollutants and those studying outdoor and indoor chemistry. Experts from these respective disciplines made presentations, each of which was followed by group discussion. At the end of the workshop the participants were charged with developing a list of research priorities and testable hypotheses at the interface between indoor chemistry and human health.

## Issues

Presentations and discussions focused on three broad issues: chemical reactions among indoor pollutants, potential health effects associated with inhalation exposure to the products of indoor chemistry, and techniques to study potential health effects. Much of what we summarize here comes from the presentations, for which we gratefully credit the presenters listed in the acknowledgments.

### Chemical reactions among indoor pollutants.

Dominant indoor processes include oxidation reactions involving oxygen, ozone, hydroxyl, and nitrate radicals; acid–base reactions, hydrolysis reactions, and decomposition reactions, often promoted by ultraviolet light and/or heat. Hydrolysis reactions are relatively slow and occur primarily on surfaces. The other processes can occur both in the gas phase and on surfaces. Characteristic times associated with air-exchange, advective transport, diffusive transport, and first-order kinetics are important for indoor-pollutant dynamics and affect the outcome of reactions.

O_3_ drives most indoor oxidative chemistry and can react at meaningful rates in the gas phase with nitric oxide (NO), nitrogen dioxide (NO_2_), and unsaturated organic compounds (e.g., terpenoids, sesquiterpenes, unsaturated fatty acids) to yield excited intermediates, OH and NO_3_ radicals, and oxygenated organic compounds (Weschler 2004). The use of cleaning products containing both terpenes and glycol ethers in the presence of O_3_ can lead to oxidation of the glycol ethers via OH and perhaps NO_3_ (Nazaroff and Weschler 2004); resultant products may include potentially allergenic peroxides and hydroperoxides (Karlberg et al. 2003). Modeling indicates that when O_3_ and NO_2_ are present simultaneously, indoor NO_3_ may be the dominant indoor oxidant; NO_3_ levels as low as 1 ppt can compete effectively with O_3_ and OH in oxidizing various terpenoids (Nazaroff and Weschler 2004). There is a need for new analytical techniques to measure the products of indoor chemistry that are short-lived, highly reactive, thermally labile, or highly oxidized—“stealth” chemicals. Oxidative chemistry has likely increased indoors over the past half-century, given increasing out-door O_3_ levels, the greater indoor use of terpenoids (as odorants and cleaning products), and decreased ventilation rates.

Surface-to-volume ratios are much larger indoors than outdoors (roughly 3 vs. 0.01 m^2^/m^3^), and consequently, surface reactions tend to be more important indoors than out. At the molecular level, the fundamental principles of surface chemistry are the same outdoors, indoors, and within the respiratory tract. Indoor surfaces are diverse, including building materials, wall cavities, ducts, skin, clothing, dust, and airborne particles. As a consequence of surface chemistry, primary species can be altered/sorbed, thereby influencing the amounts available for inhalation; many of the secondary species would not be present if indoor chemistry did not occur [e.g., products of O_3_–carpet interactions (Morrison and Nazaroff 2002; Weschler et al. 1992)]. Surface interactions influence subsequent human inhalation exposures to the constituents of environmental tobacco smoke (Nazaroff and Singer 2004); for example, acid-base chemistry influences nicotine’s desorption from surfaces (Destaillats et al. 2005). In the case of carpet emissions, the presence of O_3_ influences aldehyde emissions, with concentrations of some emitted oxidation products exceeding their odor thresholds (Morrison and Nazaroff 2002). As a consequence of sorption and re-emission from indoor surfaces, certain pesticides and fumigants that are transported indoors can remain at elevated concentrations and/or chemically transform for days or weeks. Malathion, a pesticide judged to be safe for humans, can be oxidized to malaoxon, a compound known to be toxic (Brown et al. 1993).

Other issues related to chemical reactions on surfaces include the interplay between sorption and surface reactions, the potential influence of surface chemistry on air quality in damp buildings, and the aging/“regeneration” of surfaces. One of the first examples of indoor surface chemistry was the NO_2_/surface formation of nitrous acid (HONO) and nitric acid (HNO_3_) (Pitts et al. 1985). It is now known that the resulting nitric acid on surfaces exists as an HNO_3_–H_2_O complex (Dubowski et al. 2004), yielding possible acidic, oxidizing, and nitrating surface films on interior walls. Air–water interfaces are a common feature of indoor environments, and evidence indicates that chemistry is enhanced at such interfaces. Recent molecular dynamic simulations indicate that OH can be concentrated by a factor of 6 and O_3_ by a factor of 10 at such interfaces (Roeselová et al. 2004). Similar behavior has been observed for some organic species (e.g., naphthalene). Indeed, surface chemistry within buildings may be dominated by interface reactions.

Building materials emit a myriad of reactive constituents and secondary products (derived from initial constituents). These include terpenoids, aliphatic aldehydes, phthalates, phenol, mono- and dicarboxylic acids, diisocyanates, and various photoinitiators (Salthammer et al. 2002). An example of secondary emissions occurs in houses constructed with wooden studs treated with pentachlorophenol (PCP). Over time, PCP is transformed to tetrachloroanisole, giving occupants a highly undesirable odor (Gunschera et al. 2004). So-called “ecologic” or “green” products are not necessarily free from adverse health effects; certain constituents such as terpenoids and linseed oil may be more chemically reactive than those from nonecologic products. Secondary emissions from such products may pose a greater health risk than the compounds for which their precursors are substitutes.

Thermal-desorption particle-beam mass spectrometry has identified some of the more reactive products resulting from reactions of O_3_ and NO_3_ radicals with linear and cyclic alkenes (Ziemann 2002, 2003). Many of these products are relatively unstable and would not have been detected using conventional gas chromatographic/mass spectrometric methods. Alcohols, carbonyls, and carboxylic acids enhance the formation of secondary ozonides, as well as alkoxy and acyloxy hydroperoxides, from stabilized Criegee intermediates formed in O_3_–alkene reactions (Docherty et al. 2004). In other reaction pathways, carbonyls and carboxylic acids promote peroxyhemiacetal and polymer formation. Exploration of NO_3_ radical–alkene reactions has revealed that many products are multifunctional nitroxy, carbonyl, hydroxyl, and hydroperoxyl compounds. Some of the oxidation reaction products have vapor pressures low enough to lead to increased particle formation via molecular condensation (Ziemann 2002).

There are numerous gaps in our knowledge concerning indoor reactants and their products. A current need is measurements of the concentrations of OH, NO_3_, HO_2_, and methylperoxy (CH_3_O_2_) radicals under different indoor conditions for better understanding of their indoor chemistry (Sarwar et al. 2004). Indoor chlorine and chlorine oxide (HOCl, ClO_2_) chemistry has not received much attention; emission sources for such compounds include treated tap water, bleach, and other cleaners. Anecdotal evidence exists for reactions between ClO_2_ from tap water and new carpet leading to unpleasant odors. Chemical transformations occurring within heating, ventilating, and air conditioning systems or in the immediate vicinity of the breathing zone (“near-head” chemistry) are potentially important but have been little explored. Over time, additives in consumer products undergo chemical transformations (e.g., diphthalate esters hydrolyzing to alcohols and monoesters). However, the health consequences of exposure to such transformation products are largely unknown. Additionally, the ongoing introduction of new compounds into the indoor environment necessitates continual study of indoor emissions.

### Potential health effects associated with inhalation exposure to the products of indoor chemistry.

Organic compounds routinely measured in indoor air only partially, if at all, explain irritation complaints by building occupants (Wolkoff and Nielsen 2001). There needs to be a shift from what scientists can readily measure to what truly needs to be measured to improve exposure assessments, evaluations of health impacts, and regulations. Stealth chemicals derived from indoor chemistry may be partly responsible for sensory effects (Weschler and Shields 1997; Wolkoff and Nielsen 2001). Epidemiologic studies support this hypothesis (Bluyssen et al. 1996; Sundell et al. 1993). For example, Sundell et al. (1993), in a study of 86 rooms in 29 office buildings, found that levels of “lost” total volatile organic compounds (TVOCs) (lower TVOC concentrations in the room air than in the supply air) were inversely proportional to sick-building-syndrome symptoms. The strong association between lost TVOCs and occupant symptoms provided some of the earliest evidence for an association between chemical transformations of indoor pollutants and adverse health effects.

Human sensitivity to complex mixtures of short- and long-lived radicals, ozonides, organic acids, and other oxygenated intermediates species remains unknown. Using a mouse bioassay, researchers have demonstrated that terpene oxidation products—in the O_3_/*R*-limonene, O_3_/α-pinene, and O_3_/isoprene systems—are more irritating to the upper air-ways than are terpenes or O_3_ alone (Rohr et al. 2002; Wolkoff et al. 1999). The currently identified oxidation products are insufficient to fully explain the irritation response, and unidentified oxidation products could be contributing to the effects. Short-lived species may be responsible, because the bioresponse was diminished in experiments conducted at higher relative humidity and with longer reaction times (Wilkins et al. 2003).

In one study, women in their late 20s, with no serious sensitivities, were exposed to a mixture of 40 ppb of O_3_ and 23 VOCs including two terpenes, the same mixture without O_3_, or air with a lower concentration of the VOC mixture (Fiedler et al. 2002). Monitored responses were both psychological (symptoms, odor ratings) and physiologic (lung function; neuroendocrine, neurobehavioral, and inflammatory markers). The mixture that included O_3_ had significantly higher concentrations of formaldehyde, glyoxal, hydrogen peroxide, and secondary organic aerosols. Nonetheless, participant responses were similar regardless of exposure condition. Hence, for the time scale (~ 2 hr) and sensitivity of these experiments, there was no pronounced association between exposure to the products of indoor chemistry and the effects monitored in this study.

Human eye exposures have been used as a tool for evaluating exposures to products of O_3_/alkene chemistry. No change in blink frequency (BF) was observed for O_3_ or limonene alone or the O_3_/isoprene mixture, but there was a significant increase in BF upon exposure to a mixture of O_3_/limonene or O_3_/NO_2_ (Kleno and Wolkoff 2004). Increased relative humidity decreased BF. Additional factors to examine are the role of free radicals as well as fine and ultrafine particles in blinking and eye irritation, BF response versus chemical product concentrations, the physiologic mechanisms, and the nature of chemicals that disrupt the tear film. An overarching question is whether eye blink rates provide an early warning of a health effect.

The anatomy of the upper airway and its responses to irritants such as O_3_ and chlorine are relevant to potential health effects caused by products of indoor chemistry (Shusterman 2003; Shusterman and Avila 2003). Considering the airways as a collection and filtering system designed to condition air for use by the human body allows for discrete compartmentalization based on function. The water solubility of a pollutant influences its impact on the airway: The most water-soluble chemicals affect the eyes, nose, and throat; less water-soluble chemicals affect the middle airway (bronchial tubes); and the least water-soluble chemicals affect the lower airway (deep lung and alveoli).

There is evidence that inhaled oxidant pollutants produce oxidative stress coupled with up-regulation of inflammatory cytokine production in the airways of asthmatics. Genetic polymorphisms in key antioxidant enzymes may predict susceptibility to cytotoxic tissue injury from oxidative stress (Bergamaschi et al. 2001). Reactive oxygen species found in or generated by diesel particles, fly-ash from oil furnaces, O_3_, and other oxidant air pollutants can damage lipids, proteins, and DNA and initiate a chain of events started by macrophages and targeting pollutant capture and neutralization (Arjomandi et al. 2005). Present knowledge indicates that *a*) pollutant-induced oxidative stress leads to proinflammatory gene expression through multiple pathways; *b*) oxidant pollutants can enhance responses to environmental allergens; and *c*) there are systemic effects of pollutant-induced oxidative stress in the lung that are important in cardiovascular toxicity.

O_3_ provides a good example of the consequences of inhaling a reactive pollutant. The pulmonary effects include airway hypersecretion, decreased lung function, epithelial cell damage, and inflammation. O_3_ exposure activates macrophages, the second most potent secretory cells in the body and critical mediators of inflammatory response. Macrophage overactivation, with excessive production of cytotoxic and proinflammatory mediators, can contribute to tissue injury. Mediators include cytokines, reactive oxygen intermediates such as superoxide, hydrogen peroxide, and OH radicals and reactive nitrogen intermediates (RNIs) such as NO and peroxy-nitrite (Fakhrzadeh et al. 2004; Laskin et al. 2004). Studies with O_3_-exposed rats have shown that macrophages release tumor necrosis factor-α and interleukin-18, leading, through a series of steps, to NO production and ultimately tissue injury. Blocking macrophage NO production by gadolinium chloride has been shown to prevent the observed O_3_-induced tissue injury (Pendino et al. 1995), providing evidence for RNI’s role in tissue injury. The extent to which inhaling other reactive species (e.g., peroxy radicals or hydroperoxides) results in overactivation of macrophages is not known.

Dermal exposures are also of concern. Karlberg et al. (1994) has shown that air oxidation of limonene produces contact allergens. These include limonene oxide, carvone, and a series of hydroperoxide isomers. Similarly, the oxidation of linalool yields allergenic hydroperoxide isomers (Skold et al. 2002). Special methods are required to isolate and identify hydroperoxides, which are unstable and readily form the corresponding aldehyde. When glycol ethers (ethoxylated surfactants) are exposed to air, allergenic oxidation products are also formed, although not as quickly as with terpenoids (Karlberg et al. 2003). These air-oxidation reactions are normally slow. However, some allergenic oxidation products can be formed at much faster rates through O_3_-initiated oxidation processes.

Although the workshop focused on potential acute effects that might result from exposure to the products of indoor chemistry, it was agreed that researchers should also be mindful of potential chronic effects, especially cancer.

Techniques to study potential health effects include multiple methods to study the impact of pollutants on the respiratory tract, including acoustic rhinometry, nasal peak inspiratory flow, nasal scraping, nasal lavage, olfactory testing, and trigeminal nerve sensory acuity. Physiologic changes such as watery eyes and nose or changes in the cells lining the contact surfaces can be indicators of irritation and may be quantifiable.

Biomarkers for exposure to selected products of indoor chemistry would be of obvious utility. Changes in exhaled NO (eNO) concentrations have been used to track asthma and have been associated with exposure to outdoor air pollution (Koenig et al. 2003). NO is ubiquitous in the body and is elevated in exhaled breath of asthmatics or persons having an asthma attack. Increases in fine particles and in light-absorbing carbon particles have been associated with airway inflammation, measured as increases in eNO in older subjects with asthma (although a similar increase was not observed in older subjects with chronic obstructive pulmonary disease). Given that eNO is a marker of oxidative stress, exposures to certain products of indoor chemistry (e.g., OH radicals, NO_3_ radicals, ozonides, and hydroperoxides) may also lead to increases in eNO. However, the rapid oxidation of NO by certain oxidants may complicate its utility as a biomarker.

Chemesthesis—the “feel” of a chemical, usually in the eyes, mouth, or throat—describes chemically provoked irritation. Only three receptors are involved in chemesthesis versus > 300 for olfaction. Odor perception tends to increase gradually with increasing chemical concentration, whereas chemesthesis requires a threshold concentration to elicit response and then increases fairly rapidly (Cometto-Muniz et al. 2005). Chemicals tend to stimulate at equal fractions of their saturation vapor pressure. Subjects are not able to feel chemicals with molecules above a certain size; the reason for this is not well understood. For nonreactive molecules, the chemesthesis threshold for a brief exposure is typically > 1 ppm; for reactive molecules it may be lower. In the case of a limonene/O_3_ mixture (at realistic concentrations), subjects’ chemesthesis response increased over time. The duration of the exposure has an amplifying effect on both chemesthesis magnitude and sensitivity (Cometto-Muniz et al. 2004).

A subset of building occupants is especially susceptible to pollutant exposures (Miller 1997). Such individuals can serve to alert health professionals to problematic indoor environments, including those with elevated species derived from indoor chemical reactions. There was a brief discussion at the workshop regarding methods to identify such individuals.

Workshop participants agreed that it is crucially important to understand exposures and that insufficient time had been spent discussing exposures of different populations to the products of indoor chemistry. Knowledge regarding actual exposures and intakes is extremely important in making eventual connections with health outcomes. This is an area requiring much more attention.

## Recommendations

A common theme running through workshop discussions was the need to better characterize and understand the “reacting” indoor environment, with an emphasis on the chemicals that most affect human health—the “biologically relevant” compounds. New methods need to be developed that can detect some of the elusive, short-lived, highly reactive products.

At the conclusion of the presentations, the participants were split into seven groups, each charged with developing a list of at least three research priorities and one or more hypotheses, which were subsequently discussed and prioritized by the full set of participants.

### Priority research needs.

The list of research needs generated at the workshop can be grouped into six categories (the first three were judged to be most important):

#### Exposure.

Conduct targeted exposure studies for specific compounds formed by reactions among indoor pollutants, as well as reaction product precursors. Focus on health-relevant (acute and chronic) compounds. Incorporate methods demonstrated to be useful in studies of outdoor pollutants. Take advantage of existing exposure biomarkers (or identify new biomarkers) for targeted products of indoor chemistry.

#### Modeling/measurements/model evaluation.

*a*) Evaluate indoor chemistry models by measuring the concentrations of key reaction byproducts (e.g., OH, NO_3_, HO_2_, and CH_3_O_2_ radicals) under a variety of indoor conditions. Employ existing techniques that have been successfully applied to outdoor air. Such measurements would be used to evaluate and improve the models. The improved models, in turn, would be used to focus additional measurements. Ultimately, targeted measurements of key reaction products should occur. *b*) Develop integrated pharma-cokinetic models addressing potential irritation, inflammation, and allergic responses initiated by the reaction products judged to be the most biologically significant.

#### Risk assessment.

Evaluate the health risks posed by the known products of indoor chemistry. This could be done using disability-adjusted life years [the sum of years of premature mortality plus years of illness or injury modified by appropriate weighting factors because of a particular disease or risk factor (Anand and Hanson 1997)]. Further risk assessment of reaction products would be based on toxicology, structure activity relationships, and epidemiologic studies addressing both cancer and noncancer end points.

#### Tissue irritation.

Evaluate the contribution of the products of indoor chemistry to irritation, especially mucosal irritation, and the susceptibilities of various target organs. Evaluate the consequences of chemical reactions that might occur on biologic surfaces such as skin or human lung tissue.

#### Screening test.

Develop a rapid screening test (e.g., *in vitro* cell bioassays) that would permit initial health-effects evaluation of compounds generated by reactions among indoor pollutants.

#### Integrated program addressing inflammation, allergies, and asthma.

Screen products of indoor chemistry for their potential to exacerbate allergies or asthma and irritate mucous membranes. After screening, evaluate the public’s exposures to the compounds of greatest concern coupled with detailed evaluations of these compounds’ toxicology.

### Testable hypotheses.

The participants agreed that the subject of the workshop itself could be stated as a testable hypothesis—that products of indoor chemistry adversely affect human health. More specifically, the testable hypotheses offered by the participants covered four areas:

- Mucosal irritation: chemical transformations of indoor pollutants yield products that contribute to mucosal irritation and inflammation.
- Allergies: selected products of indoor chemistry can promote allergies (type 1 hypersensitivity).
- Intervention: removing O_3_ or sources of chemically reactive pollutants will lead to health improvements in environments where the intervention occurs (by limiting the products of O_3_-initiated chemistry).
- Ecologic labels: chemical transformations of constituents found in various indoor “green” or “ecologic” materials subsequently contribute to, rather than mitigate, health problems.

The focused research needs identified at the Indoor Chemistry and Health workshop are consistent with the broader research needs identified in the 2002 NORA indoor environment team publication (Mendell et al. 2002).

## Conclusions

In the developed world, human exposure to airborne chemicals is dominated by indoor exposures. Inhalation of airborne pollutants is known to adversely affect human health, producing both acute and chronic effects. These include mucous membrane irritation, allergies and asthma, cardiopulmonary effects, and cancer. Some of the species inhaled indoors come from outdoors; some come directly from materials and products used indoors, and some are a consequence of chemical reactions occurring in the indoor environment. Certain chemical processes are continually occurring indoors (e.g., hydrolysis of esters on indoor surfaces). Other chemical processes are occurring intermittently, varying with time of day, day of week, season, and location (e.g., O_3_-initiated oxidation of terpenoids). Discussions throughout the workshop made it clear that research designed to evaluate the potential impacts that such indoor chemical processes have on human health has only just begun. The challenges in this emerging field are to define exposures to products of indoor chemistry and develop an understanding of the links between these exposures and various health outcomes. For this to happen, health professionals and associated researchers must be aware of indoor chemistry. The Indoor Chemistry and Health workshop was an early step in developing such awareness.
